# Ferrous sulfate induces ferroptosis-like cell death in *Trichosporon asahii*

**DOI:** 10.3389/fmicb.2026.1789479

**Published:** 2026-04-01

**Authors:** Lingzhi Xu, Haochen Guo, Xin Yang, Rongya Yang, Zhikuan Xia

**Affiliations:** 1 Zhujiang Hospital, The Second School of Clinical Medicine, Southern Medical University, Guangzhou, China; 2 Department of Dermatology, The Seventh Medical Center of Chinese PLA General Hospital, Beijing, China; 3 Chinese PLA Medical School, Beijing, China

**Keywords:** ferroptosis, ferrous sulfate, lipid peroxidation, membrane damage, reactive oxygen species, transcriptomics, *Trichosporon asahii*

## Abstract

*Trichosporon asahii* is a significant pathogen of invasive fungal infections, exhibiting low susceptibility to multiple first-line antifungal drugs, which poses severe challenges in clinical treatment. This study discovered for the first time that ferrous sulfate (FeSO₄) has a potent inhibitory effect against this fungus by inducing a ferroptosis-like cell death. This process ischaracterized by non-apoptotic cell death, iron ion dependence, reactive oxygen species (ROS) burst, lipid peroxide accumulation, and damage to key membrane systems including the loss of mitochondrial membrane potential. This process can be partially reversed by the ferroptosis-specific inhibitor Ferrostatin-1, and provided a modest improvement in fungal survival. Transcriptomic analysis further revealed that FeSO₄ treatment causes significant alterations in the expression of genes related to redox processes, glutathione metabolism, and the proteasome pathway. This study, for the first time, elucidates a ferroptosis-like death pathway in *Trichosporon asahii* triggered by exogenous Fe^2+^, which depends on iron accumulation and lipid peroxidation, thereby revealing a novel, ferroptosis-based mechanism with potential for future antifungal development.

## 1 Background

Invasive fungal infections refer to severe infections caused by pathogenic fungi invading deep human tissues or organs, characterized by complex clinical manifestations and high mortality rates (Chagas-Neto et al., 2008). Over the past two decades, the incidence of *Trichosporon* infections has shown a yearly increasing trend (Liao et al., 2015). *T. asahii* is the most prominent clinical pathogen in the genus, causes more than half of the infections (Makinodan et al., 2005). It can cause a spectrum of infections, ranging from superficial cutaneous or mucosal involvement to life-threatening systemic dissemination (Francisco et al., 2021). A systematic review of 140 cases between 1996 and 2019 found that *Trichosporon asahii* infections are predominantly reported in Asia, accounting for 77.1% of cases, with a notable increase observed from 2006 to 2015. The infection mainly affects immunocompromised individuals with underlying conditions such as hematological diseases, diabetes, or pulmonary diseases, most commonly presenting as urinary tract infection, fungemia, or disseminated infection. The overall mortality was 30.0%, with 13.6% of deaths directly attributed to the infection (Li et al., 2020). This pathogen exhibits low susceptibility to first-line antifungal drugs such as amphotericin B and is intrinsically resistant to echinocandins, making clinical treatment extremely challenging (Mehta et al., 2021). Currently, triazole drugs are the first-line choice for the prevention and treatment of systemic trichosporonosis. However, their widespread use has accelerated the emergence of resistant strains, even leading to multidrug resistance, which further limits therapeutic options (Montoya et al., 2015; Kalkanci et al., 2010). A systematic analysis by Liao et al. showed that among 185 patients with trichosporonemia, 80 experienced breakthrough infections, most of which were associated with the use of amphotericin B and echinocandins (Liao et al., 2015). In contrast, azole drugs demonstrate significant anti-*Trichosporon* activity both *in vitro* and *in vivo*, and clinical data support their advantage in improving patient outcomes (Li et al., 2020; Ramírez and Moncada, 2020). The 2021 global guideline for rare yeast infections recommends fluconazole and voriconazole as initial treatment drugs, with voriconazole as the preferred choice (Chen et al., 2021; Sprute et al., 2022). However, the widespread use of azole drugs has inevitably driven the evolution of resistance. Therefore, exploring novel antifungal strategies that act beyond traditional targets has become an urgent task in current research.

Ferroptosis is a form of non-apoptotic cell death characterized by lipid peroxidation and dependent on iron and reactive oxygen species, first proposed by the team of Brent R. Stockwell in 2012 (Dixon et al., 2012). In recent years, ferroptosis has attracted widespread attention due to its potential in disease treatment (Jiang et al., 2015; Berndt et al., 2024; Xu et al., 2023; Hassannia et al., 2019). Ferroptosis can be triggered by extrinsic pathways involving drugs, small molecule compounds, hypoxia, and radiation (Tang and Kroemer, 2020; Pinochet-Barros and Helmann, 2018). It is a regulated form of cell death associated with the accumulation of lipid peroxidation markers and results from lethal lipid peroxidation in mammalian cells (Stockwell et al., 2017). Cell death induced by ferroptosis can be inhibited by iron chelators, lipid peroxidation inhibitors, liposoluble antioxidants, and depletion of polyunsaturated fatty acids (Stockwell et al., 2017). Although initially identified and extensively studied in mammalian cells, ferroptosis-like phenomena have also been increasingly observed in fungi. Research has clearly documented the existence and biological functions of ferroptosis in fungal cells, such as in Magnaporthe oryzae (Gu et al., 2025), *Candida albicans* (Miao et al., 2025).

Given the ubiquity of iron in biological systems, ferroptosis is considered a conserved cell death mechanism across species, prevalent in various organisms including fungi (Conrad et al., 2018; Kulkarni et al., 2019). The regulatory mechanisms of fungal ferroptosis, while in nascent stages, may combine conserved eukaryotic ferroptosis pathways with fungal-specific metabolic characteristics (Shang et al., 2025). Iron metabolism regulation is central to this process; fungi possess various iron uptake systems, and when iron ions are excessively taken up, they can initiate the ferroptosis program. Specifically, Fe^2+^ participates in ferroptosis through two key reactions: the Fenton reaction, generating highly reactive hydroxyl radicals that attack polyunsaturated fatty acids (PUFAs) in the cell membrane; and acting as a cofactor for lipoxygenases (LOX), catalyzing the oxidation of PUFAs to generate lipid peroxides. As a natural and safe compound playing a crucial role in fungal growth and metabolism, iron is considered to have the potential for developing therapeutic agents that reduce drug resistance and enhance efficacy (Chagas-Neto et al., 2008; Montoya et al., 2015). Given that iron is the core element triggering ferroptosis, direct treatment with iron compounds is regarded as a straightforward approach to induce this form of cell death. Studies have shown that iron can induce ferroptosis-like death in various pathogenic microorganisms, such as *Staphylococcus aureus* (Wang et al., 2022; Zhao et al., 2022), *Aspergillus flavus* (Yao et al., 2021), and *Candida albicans* (Miao et al., 2025). However, whether iron induces ferroptosis in *Trichosporon asahii* and its specific mechanisms remain unknown.

Therefore, this study employed ferrous sulfate to induce iron overload in Trichosporon asahii, in order to systematically investigate the occurrence of ferroptosis and its underlying molecular mechanisms. The research focuses on elucidating the pathway through which exogenous iron triggers ferroptosis-like cell death in this fungus. This work aims to explore the potential of inducing ferroptosis as a novel mechanism for future antifungal development.

## 2 Experimental methods

### 2.1 Strain and culture conditions

The *Trichosporon asahii* type strain CBS 2479 was purchased from the CBS-KNAW Fungal Biodiversity Centre, Utrecht, The Netherlands. Ten clinical isolates (BMT 06-3-01 to BMT 06-3-10) were provided by the Dermatology Department of a collaborating hospital and were identified by ribosomal DNA internal transcribed spacer (ITS) sequencing. The antifungal susceptibility profiles of all 11 strains, including CBS 2479, are provided in Supplementary Table S1. All strains were cultured on Potato Dextrose Agar (PDA) medium at 37 °C in the dark. For experimental use, strains were inoculated into YPD liquid medium and incubated at 37 °C with shaking at 180 rpm for 24 h. Cells from the logarithmic growth phase were collected, washed twice with PBS (pH 7.2), resuspended, and adjusted to a concentration of 1 × 10^6^ CFU·mL^−1^. All 11 strains were included in the preliminary antifungal screening of iron compounds. Given the consistent inhibitory effects observed across all strains, subsequent mechanistic studies were carried out using the type strain CBS 2479. Ferrous sulfate hexahydrate (FeSO₄·6H₂O) was purchased from Shanghai Hushi Reagent Co., Ltd. All other analytical grade chemical reagents were purchased from Sigma-Aldrich (United States).

### 2.2 Antifungal susceptibility testing

#### 2.2.1 Comparison of iron salt antimicrobial activity and survival rate determination

To systematically evaluate the antimicrobial activity of different exogenous iron compounds against *Trichosporon asahii*, FeSO₄·6H₂O, Fe₂(SO₄)₃·xH₂O FeCl₂·4H₂O, FeCl₃, and Fe-EDTA were used in this study. All iron compound solutions were freshly prepared in ultrapure water before each experiment, with precautions taken to minimize exposure to air during preparation. Each compound was then diluted in YPD medium to prepare a final concentrations of 1, 2, 5, and 10 mM to evaluate their antifungal activity against *T. asahii*. A microdilution method was employed: 100 μL of drug-containing medium and 100 μL of the fungal suspension (1 × 10^6^ CFU·mL^−1^) were added to each well of a 96-well plate, followed by incubation at 37 °C with shaking at 150 rpm for 24 h. Subsequently, the culture was serially diluted 10-fold with sterile PBS, and 100 μL was spread onto PDA plates. After incubation at 37 °C for 24 h, colonies were counted. The survival rate was calculated as: Survival rate (%) = (CFU of experimental group / CFU of blank control group) × 100%. Data were plotted as concetration of iron salt versus survival percentage, and non-linear regression analysis was performed using GraphPad Prism (v9.0) with a four-parameter logistic model to calculate the half-maximal inhibitory concentration (IC₅₀). Ferrous sulfate hexahydrate (FeSO₄·6H₂O) and Fe₂(SO₄)₃·xH₂O was purchased from Shanghai Hushi Reagent Co., Ltd. FeCl₂·4H₂O was purchased from Macklin Reagent Co., Ltd. All other analytical grade chemical reagents were purchased from Sigma-Aldrich (United States).

#### 2.2.2 Exclusion of VBNC state

After treatment with 5 mM FeSO₄ for 6, 12, 24, 48, and 72 h, 1 mL of fungal suspension (~10^6^ cells) was stained with 100 μL of 5 μg·mL^−1^ propidium iodide (PI, MedChemExpress, United States) in the dark at room temperature for 10 min. The samples were then centrifuged at 6,000 g for 5 min, the supernatant was discarded, and the pellet was resuspended in 1 mL PBS and stained with 100 μL of 2 μg·mL^−1^ DAPI in the dark for 15 min. The stained samples were directly observed under a fluorescence microscope without further centrifugation.

For fluorescence intensity quantification, fungal suspensions treated with 5 mM FeSO₄ for 24 h were stained with 5 μg·mL^−1^ PI in the dark at room temperature for 10 min. Fluorescence intensity was measured using a multifunctional microplate reader (Spark, Tecan, Switzerland) with excitation/emission wavelengths set at 535/617 nm according to the manufacturer’s instructions. All samples were placed in black 96-well plates (200 μL per well) and shaken for 3 min before reading. Morphological observations were performed using an inverted fluorescence microscope (DMi8, Leica, Germany). Images were acquired using LAS X software (version 3.7.4) and analyzed using ImageJ software (version 1.53).

For quantitative analysis, ≥3 random fields (≥200 cells total) were imaged per sample using a fluorescence microscope (DMi8, Leica, Germany), and the percentage of PI-positive cells was calculated. Parallel untreated controls were included at all time points to monitor culture senescence. Simultaneous plate colony counts were performed. The methodology was synthesized from several previous studies (Li et al., 2020; Fleischmann et al., 2021).

### 2.3 Morphological observation

A spore suspension at a concentration of 1 × 10^6^ cells·mL^−1^ was exposed to 5 mmol·L^−1^ FeSO₄ at 37 °C with shaking at 150 rpm for 12 h. After centrifugation and removal of the supernatant, electron microscopy fixative was added, and fixation was performed in the dark at room temperature for 2 h, followed by storage at 4 °C. Samples were dehydrated through a graded ethanol series (30–100%, 10 min per concentration), dried under vacuum, mounted on stubs with conductive adhesive, and sputter-coated with a metal film (gold or platinum) approximately 50–300 Å thick. Cell morphology and surface structure were observed and recorded using a scanning electron microscope (TM3000, Hitachi, Japan).

### 2.4 Investigation of ferroptosis mechanism

#### 2.4.1 Reagents

The ferroptosis inhibitors Ferrostatin-1 (Fer-1, 20 μM) and Liproxstatin-1 (Lip-1, 10 μM), the reactive oxygen species scavenger N-acetylcysteine (NAC, 5 mM), reduced glutathione (GSH, 5 mM), the iron chelator 2,2′-bipyridyl (Dipy, 100 μM), the apoptosis inhibitor Z-VAD-FMK (20 μM), the cell death inhibitor cycloheximide (CHO, 20 μM) and the necroptosis inhibitor Nec-1 (50 μM) were all purchased from MedChemExpress (United States). The concentrations of the inhibitors used were determined either by referencing concentrations proven effective in previous related studies (Fer-1, Lip-1, NAC, GSH, Dipy) (Mo et al., 2024) (Wang et al., 2022) (Han et al., 2012) through pre-experiments conducted in this study.

#### 2.4.2 Cell survival rate under inhibitor intervention

Fungal cells were co-incubated with 2 mM FeSO₄ or 5 mM FeSO₄ and the aforementioned inhibitors for 12 h to sensitively monitor inhibitor rescue during active cell death. Subsequently, a 10-fold serial dilution was performed using sterile PBS, and 100 μL was spread onto PDA plates. After incubation at 37 °C for 24 h, colonies were counted. The relative survival rate of each inhibitor group was calculated, with the untreated control group set as 100%.

#### 2.4.3 Intracellular Fe^2+^, ROS, and lipid peroxidation detection

Intracellular reactive oxygen species (ROS) were detected using the Reactive Oxygen Species Assay Kit (S0033S, Beyotime, China), which employs the fluorescent probe DCFH-DA. Lipid peroxidation was assessed using the Lipid Peroxidation Assay Kit (S0039, Beyotime, China), which utilizes C11-BODIPY 581/591. Intracellular ferrous iron (Fe^2+^) was detected using Ferrous Ion Fluorescent Probe (Elabscience). Following treatment with FeSO₄, a *T. asahii* suspension (1 × 10^6^ CFU·mL^−1^) was incubated with the respective probe at the following working concentrations: 10 μM DCFH-DA, 2 μM C11-BODIPY 581/591, or 5 μM Ferrous Ion Fluorescent Probe (Elabscience) Fluorescence intensity was detected using a Tecan Spark multifunctional microplate reader (DCFH-DA: Ex/Em = 488/525 nm; C11-BODIPY: Ex/Em = 488/510 nm; Ferrous Ion Fluorescent Probe: Ex/Em = 542/580 nm). Simultaneously, 200 μL of the cell suspension was placed in a black 96-well plate for synchronous imaging verification using an inverted fluorescence microscope (DMi8, Leica, Germany). Microscope images were acquired using LAS X software (version 3.7.4) and analyzed using ImageJ software (version 1.53).

#### 2.4.4 Malondialdehyde content determination

Total protein was extracted using RIPA lysis buffer (containing 1 mM EDTA). The Malondialdehyde (MDA) content was determined according to the instructions of the Beyotime (Shanghai) MDA detection kit.

#### 2.4.5 Mitochondrial membrane potential (ΔΨm) detection

JC-1 dye (5 μg·mL^−1^, Beyotime) was added to the cell suspension and incubated at 37 °C in the dark for 20 min. Multifunctional microplate reader (Tecan, Switzerland) was used to detect red fluorescence (Ex/Em = 535/590 nm) and green fluorescence (Ex/Em = 485/530 nm), and the red/green fluorescence ratio was calculated. Simultaneously, 200 μL of the cell suspension was placed in a black 96-well plate for synchronous imaging using an inverted fluorescence microscope (DMi8, Leica, Germany). Microscope images were acquired using LAS X software (version 3.7.4) and analyzed using ImageJ software (version 1.53).

#### 2.4.6 Nile red staining assay

*T. asahii* suspension (1 × 10^6^ CFU·mL^−1^) treated with FeSO₄ was incubated with Nile Red (1 μg·mL^−1^, MCE, United States) at 37 °C in the dark for 20 min, followed by a gentle wash with PBS. Fluorescence intensity was measured using a Multifunctional microplate reader (Tecan, Switzerland) (Ex/Em = 485/590 nm), and imaging was performed at the same wavelength using an inverted fluorescence microscope (DMi8, Leica, Germany) to observe intracellular neutral lipid accumulation and changes in membrane permeability. Microscope images were acquired using LAS X software (version 3.7.4) and analyzed using ImageJ software (version 1.53).

### 2.5 Transcriptomic analysis

*Trichosporon asahii* cells exposed to 5 mM FeSO₄ were sampled after 12 h of treatment, rapidly frozen in liquid nitrogen, and ground. Total RNA was extracted using Trizol reagent (Invitrogen, United States). RNA integrity was assessed by 1% agarose gel electrophoresis and an Agilent 2100 Bioanalyzer, ensuring RNA Integrity Number (RIN) values > 7.0. Qualified samples were used for cDNA library construction using the Hieff NGS® Ultima Dual-mode mRNA Library Prep Kit (Yeasen, Cat# 12309ES) according to the manufacturer’s instructions. The constructed libraries were sequenced on a BIG T7 platform (BGI) with paired-end 150 bp (PE150) reads, aiming for a sequencing depth of 6 Gb per sample. Each treatment group included three biological replicates, and no additional control samples were included in this run.

For bioinformatics analysis, raw reads were first assessed for quality using fastp (v0.23.2) to remove adapter sequences, reads with >10% N bases, reads consisting entirely of A bases, and low-quality reads (where >50% of bases had *Q* ≤ 20). Clean reads were then aligned to a ribosomal RNA database using bowtie2 (v2.4.5) to remove rRNA contamination. The remaining reads were mapped to the reference genome of *Trichosporon asahii* (ASM27752v1, NCBI Assembly ID: GCA_000277525.2) using HISAT2 (v2.2.1) with default parameters. Transcript assembly and quantification were performed with Stringtie (v2.2.1) and RSEM (v1.3.3), respectively, to generate gene-level read counts.

Differential expression analysis was conducted using edgeR (or DESeq2; version to be specified). Read counts were normalized using the TMM method (edgeR) or median ratio method (DESeq2). Statistical testing was based on a negative binomial model, and resulting *p*-values were adjusted for multiple testing using the Benjamini–Hochberg false discovery rate (FDR). Genes with |log₂(Fold Change)| > 1 and FDR < 0.05 were considered differentially expressed.

For functional enrichment, GO and KEGG annotations were retrieved from the Gene Ontology database^1^ and the KEGG Pathway database, respectively (database versions to be specified). Enrichment analysis was performed using clusterProfiler (version to be specified) based on the hypergeometric test, with FDR correction (Benjamini–Hochberg). Terms/pathways with FDR-adjusted *p*-value (*q*-value) < 0.05 were regarded as significantly enriched. Visualization included volcano plots for DEGs and bar or dot plots for enriched GO terms and KEGG pathways.

The raw transcriptome sequencing data supporting the findings of this study have been deposited in the NCBI database and are now accessible under the BioProject accession number PRJNA1432677.

### 2.6 Statistical analysis

All experiments were independently repeated three times, and data are presented as mean ± standard deviation (mean ± SD). Comparisons among multiple groups were performed using one-way analysis of variance (ANOVA) with GraphPad Prism 9.0 (United States) software, followed by Tukey’s *post-hoc* test. A *p*-value of less than 0.05 was considered statistically significant.

## 3 Results

### 3.1 Ferrous sulfate significantly inhibits the *in vitro* proliferation of *Trichosporon asahii*

**Figure 1:**
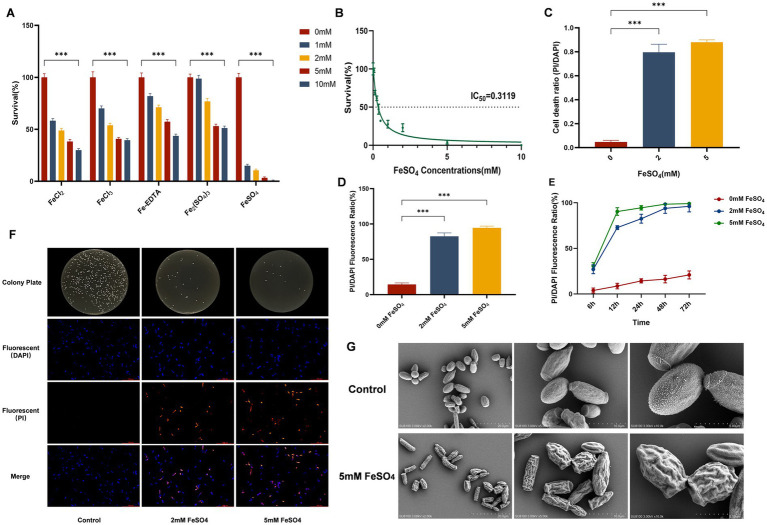
Antifungal effect of ferrous sulfate (FeSO_4_) against *Trichosporon asahii*. **(A)** Cell survival rates after treatment with different iron compounds (FeSO_4_, Fe_2_(SO_4_)_3_, FeCl_2_, FeCl_3_, Fe-EDTA) at the indicated concentrations (0–10 mM) for 24 h. **(B)** Dose–response curve and IC_50_ value of FeSO_4_ treatment for 24 h. **(C)** Ratio of PI/DAPI-positive cells after treatment with 5 mM FeSO_4_ for 24 h, quantified by fluorescence microscopy (≥3 random fields, ≥200 cells). **(D)** PI/DAPI fluorescence intensity ratio after treatment with 5 mM FeSO_4_ for 24 h, measured by a microplate reader (PI Ex/Em = 535/617 nm; DAPI Ex/Em = 358/461 nm). **(E)** PI/DAPI fluorescence intensity ratio after treatment with 5 mM FeSO_4_ for 6–72 h, measured by a microplate reader. **(F)** PDA plate colony growth (top) and corresponding fluorescence microscopy images of PI/DAPI staining (bottom, PI: red, DAPI: blue) after treatment with 5 mM FeSO_4_ for 24 h. **(G)** Scanning electron microscopy images of the control group and cells treated with 5 mM FeSO_4_ for 12 h. All quantitative data are presented as mean ± SD from three independent experiments (*n* = 3). Statistical significance was determined by one-way or two-way ANOVA with appropriate *post-hoc* tests, with significance indicated as *p < 0.05, **p < 0.01, ***p < 0.001 compared to the control group.

To evaluate the antimicrobial activity of different iron compounds against *Trichosporon asahii*, this study compared the inhibitory effects of various iron salts. The results showed that at the same concentration, ferrous sulfate (FeSO₄) exhibited significantly superior antimicrobial activity compared to other compounds. A concentration of 10 mM FeSO₄ was sufficient to kill 99.99% of the fungal cells (Figure 1A). To quantitatively compare the antifungal potency of different iron compounds, we calculated their IC_50_ values based on the dose–response curves (Figure 1B). FeSO₄ exhibited the lowest IC_50_ value (0.3119 mM), further confirming its superior antifungal activity (Supplementary Figure S1). Concentration-response curve further indicated that 2 mM FeSO₄ significantly inhibited the growth of *T. asahii*, while a 5 mM concentration almost completely blocked its proliferation (Figure 1B). To confirm whether FeSO₄-induced cell death belonged to a “viable but non-culturable” (VBNC) state, we combined colony counting with PI/DAPI staining for analysis. The results showed a increase in the proportion of PI/DAPI-positive cells in the FeSO₄-treated group, which was consistent with the colony count data (Figures 1C,D,F). Furthermore, time-course monitoring over 6–72 h revealed a sustained increase in the PI/DAPI ratio in the treated group, whereas the untreated control group exhibited a slight increase, remaining far below that of the treated group throughout the experiment (Figure 1E). This rules out the influence of culture senescence. Collectively, these findings indicating that FeSO₄ caused genuine cell death rather than a VBNC state.

### 3.2 Ferrous sulfate causes morphological and ultrastructural damage

Scanning electron microscopy observations revealed that FeSO₄ treatment caused severe damage to the cellular structure of *T. asahii*. As shown in Figure 1G, compared to the control group, treated cells exhibited obvious morphological abnormalities such as cell wall collapse, surface shrinkage, and loss of membrane integrity, suggesting severe structural damage.

### 3.3 The ferroptosis inhibitor Fer-1 reverses the proliferation inhibition induced by ferrous sulfate

**Figure 2:**
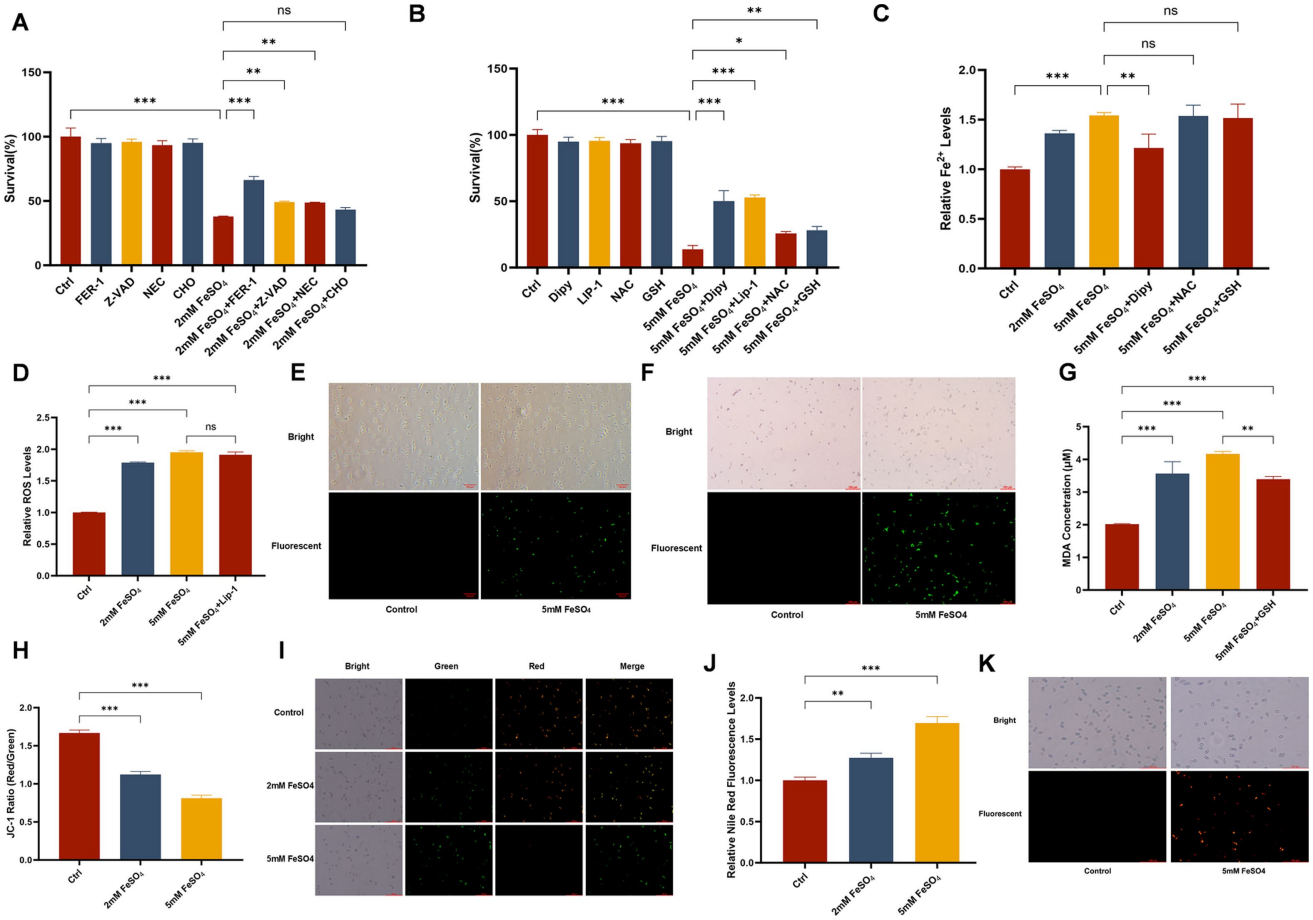
Mechanisms of FeSO_4_-induced killing in *Trichosporon asahii*. **(A)** Cell survival rates after treatment with FeSO_4_ (2 mM, 12 h) combined with different cell death pathway inhibitors. Inhibitor concentrations: ferroptosis inhibitor Fer-1 (20 μM), apoptosis inhibitor Z-VAD-FMK (20 μM), necroptosis inhibitor Nec-1 (50 μM), and cell death inhibitor CHO (20 μM). **(B)** Cell survival rates after treatment with FeSO_4_ (2 mM, 12 h) combined with ferroptosis-related inhibitors. Inhibitor concentrations: Fer-1 (20 μM), Lip-1 (10 μM), NAC (5 mM), GSH (5 mM), and Dipy (100 μM). **(C)** Relative intracellular Fe^2+^ levels (ferrous ion fluorescent probe, Ex/Em = 542/580 nm). **(D)** Relative intracellular ROS levels (DCFH-DA probe, Ex/Em = 488/525 nm). **(E)** Representative fluorescence microscopy images of ROS staining (same as **D**). **(F)** Lipid peroxidation level (C11-BODIPY 581/591 probe, Ex/Em = 488/590 nm). **(G)** Malondialdehyde (MDA) content (μM). **(H)** JC-1 red/green fluorescence intensity ratio (Ex/Em = 535/590 nm and 485/530 nm). **(I)** Representative fluorescence microscopy images of JC-1 staining (red: aggregated form, high ΔΨm; green: monomeric form, low ΔΨm). **(J)** Relative Nile Red fluorescence intensity (Ex/Em = 485/590 nm). **(K)** Representative fluorescence microscopy images of Nile Red staining (neutral lipids). All quantitative data are presented as mean ± SD from three independent experiments (*n* = 3). FeSO_4_ was used at 2 mM for 12 h **(A,B)** or 24 h **(C–J)**. Statistical significance was determined by one-way ANOVA with Dunnett’s or Tukey’s *post-hoc* tests, with significance indicated as *p < 0.05, **p < 0.01, ***p < 0.001 (compared to the control group or as specified).

To investigate the mechanism of FeSO₄-induced cell death, we employed various inhibitors of cell death pathways. The results showed that the ferroptosis-specific inhibitor Ferrostatin-1 (Fer-1) most significantly reversed the proliferation inhibition caused by FeSO₄ (Figure 2A), preliminarily suggesting a key role for a ferroptosis-like pathway in this process.

### 3.4 Ferrous sulfate promotes Fe^2+^ influx and intracellular accumulation

Detection using the FeRhoNox-1 fluorescent probe revealed that exogenous FeSO₄ treatment significantly promoted Fe^2+^ influx. The fluorescent signal was primarily localized intracellularly, indicating that Fe^2+^ could rapidly cross the membrane and accumulate within the cells (Figure 2C). The iron chelator 2,2′-bipyridyl (Dipy) partially attenuated FeSO₄-induced intracellular Fe^2+^ accumulation (Figure 2C). In addition, co-treatment with Dipy resulted in a partial rescue of cell viability (Figure 2B), demonstrating that FeSO₄-induced cell death is dependent on Fe^2+^ influx and accumulation. Furthermore, treatment with the ROS scavenger NAC or the lipid peroxidation inhibitor GSH did not reduce intracellular Fe^2+^ levels (Figure 2C), further confirming that iron accumulation is an upstream event in this death pathway.

### 3.5 Intracellular iron accumulation triggers a reactive oxygen species burst

Using the DCFH-DA fluorescent probe to detect intracellular reactive oxygen species (ROS) levels, it was found that FeSO₄ treatment caused a sharp increase in ROS within *T. asahii* cells (Figure 2D), with a subset of cells exhibiting strong green fluorescence (Figure 2E), indicating that Fe^2+^ influx triggered a significant ROS burst. Concurrently, NAC treatment partially rescued the survival rate of the fungus exposed to FeSO₄, improving survival approximately 20% (Figure 2B). To explore the main source of ROS, we intervened using Liproxstatin-1 (Lip-1), an inhibitor of the Fenton reaction. The results showed that Lip-1 could reverse the killing effect of FeSO₄ on *T. asahii*, increasing survival to about 50% (Figure 2B). However, Lip-1 did not significantly reduce intracellular ROS levels compared to FeSO₄ treatment alone (Figure 2E). This suggests that, in addition to the classical Fenton reaction, other pathways might also contribute to ROS accumulation.

### 3.6 ROS burst induces lipid peroxidation and membrane system damage

Using the C11-BODIPY probe to assess lipid peroxidation levels, it was found that FeSO₄ could induce lipid peroxidation in *T. asahii* (Figure 2F). The results of malondialdehyde (MDA) content determination were consistent with this, showing a significant increase in MDA levels in the treated group (Figure 2G). The lipid peroxidation inhibitor GSH partially alleviated this phenomenon and improved cell survival (Figure 2B).

Further mechanistic studies indicated that lipid peroxidation directly led to damage in key membrane systems. JC-1 staining revealed that FeSO₄ treatment caused a significant decrease in mitochondrial membrane potential (Figures 2H,I), indicating impaired mitochondrial function. Nile Red staining showed marked accumulation of neutral lipids in fungal cells (Figures 2J,K). These results collectively demonstrate that lipid peroxidation ultimately disrupts the integrity of cellular membrane systems, serving as a key terminal event driving cells toward ferroptosis-like death.

### 3.7 Transcriptomic analysis

**Figure 3:**
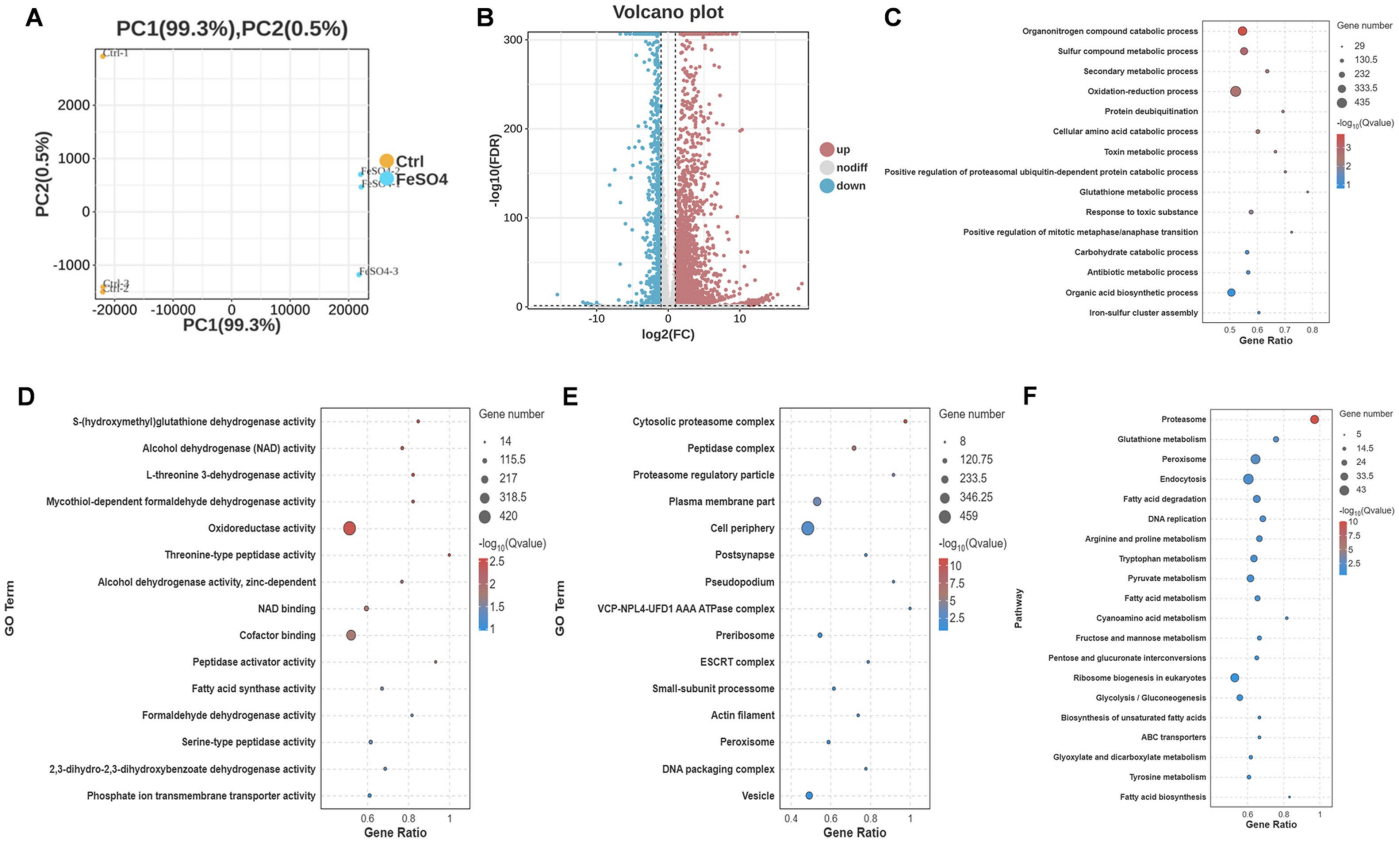
Impact of FeSO_4_ treatment on the transcriptome of *Trichosporon asahii* and functional enrichment analysis. **(A)** Principal component analysis (PCA) plot of samples from the control and FeSO_4_-treated groups (5 mM FeSO_4_, 12 h, *n* = 3 per group). **(B)** Volcano plot of differentially expressed genes (DEGs), showing the distribution of upregulated and downregulated genes. DEGs were defined as |log₂FC| > 1 and FDR < 0.05 (edgeR/DESeq2 with TMM/median ratio normalization). **(C)** Gene ontology (GO) enrichment analysis of DEGs in the biological process (BP) category. **(D)** GO enrichment analysis of DEGs in the molecular function (MF) category. **(E)** GO enrichment analysis of DEGs in the cellular component (CC) category. **(F)** Kyoto encyclopedia of genes and genomes (KEGG) pathway enrichment analysis of DEGs. For GO and KEGG enrichment analyses, significance was determined by hypergeometric test with Benjamini-Hochberg correction (FDR < 0.05). All data are from three independent biological replicates (*n* = 3).

To further elucidate the mechanism by which ferrous sulfate induces ferroptosis in *Trichosporon asahii*, this study employed RNA sequencing technology to comprehensively analyze changes in its transcriptome. Principal component analysis (PCA) showed a clear separation between the control and treatment groups in their transcriptional profiles, indicating significant inter-group differences (Figure 3A).

Using a false discovery rate (FDR) < 0.05 and |log2 (fold change)| ≥ 1 as the criteria for screening differentially expressed genes (DEGs), a total of 3,799 DEGs were identified (out of 8,729 total annotated genes, representing 43.52% of the transcriptome). Among these, 2,766 genes were upregulated and 1,033 genes were downregulated (Figure 3B), demonstrating that FeSO₄ exerts extensive and significant regulatory effects on the gene expression network of *T. asahii*. Table 1 lists the 20 most significantly upregulated and downregulated genes based on fold change. Notably, among the differentially expressed genes are several related to oxidative stress response such as genes encoding glutathione peroxidase and glutathione S-transferase, and iron ion homeostasis such as the gene encoding the high-affinity iron permease FTR1 (Table 1). This suggests that iron stress triggers extensive cellular defense and metabolic reprogramming. Due to the relatively low level of genome annotation for *Trichosporon asahii*, 70% of the Top 20 DEGs lack clear functional annotation. The annotation information of all DEGs is available in Supplementary Table S2.

**Table 1:** Top 20 DEGs in *Trichosporon asahii* responding to FeSO4.

Gene ID	log2(fc)	Symbol	Description	Regulation
ncbi_25988136	18.21	A1Q1_04624	Uncharacterized protein	Up
ncbi_25987216	15.29	A1Q1_03703	Uncharacterized protein	Up
ncbi_25987318	14.62	A1Q1_03805	Uncharacterized protein	Up
ncbi_25989216	14.30	A1Q1_05704	Alpha-galactosidase	Up
ncbi_25983836	14.12	A1Q1_00322	Extracellular membrane protein CFEM domain-containing protein	Up
ncbi_25991338	13.90	A1Q1_07826	Uncharacterized protein	Up
ncbi_25987157	13.88	A1Q1_03644	Uncharacterized protein	Up
ncbi_25984694	13.78	A1Q1_01180	Cyanovirin-N domain-containing protein	Up
ncbi_25986728	13.77	A1Q1_03215	Uncharacterized protein	Up
ncbi_25986786	13.63	A1Q1_03273	Beta-lactamase-related domain-containing protein	Up
ncbi_25986586	−15.4930319	A1Q1_03073	Uncharacterized protein	Down
ncbi_25986620	−11.91886324	A1Q1_03107	Uncharacterized protein	Down
ncbi_25986813	−11.57443585	A1Q1_03300	Carbohydrate-binding module family 13 protein/putative endo-1,3-beta-glucanase	Down
ncbi_25988357	−11.29423782	A1Q1_04845	Uncharacterized protein	Down
ncbi_25986621	−11.03754695	A1Q1_03108	Uncharacterized protein	Down
ncbi_25986388	−10.60208021	A1Q1_02875	Uncharacterized protein	Down
ncbi_25987150	−10.18115226	A1Q1_03637	Uncharacterized protein	Down
ncbi_25986593	−9.997649951	A1Q1_03080	SH3 domain-containing protein	Down
ncbi_25984032	−9.630570499	A1Q1_00518	Uncharacterized protein	Down
ncbi_25989528	−9.368506462	A1Q1_06016	Uncharacterized protein	Down

### 3.8 Functional enrichment analysis

Gene Ontology (GO) enrichment analysis was performed on the DEGs, covering Biological Process (BP), Molecular Function (MF), and Cellular Component (CC) categories. In the BP category (Figure 3C), significantly enriched terms were primarily related to protein degradation processes (GO:0010498, GO:1903364, GO:0016579), detoxification metabolism (GO:0098754, GO:0009404, GO:0010127), and redox processes (GO:0055114). Other significant BP terms included nitrogen compound catabolism (GO:1901565) and sulfur compound metabolism (GO:0006790). In the MF category (Figure 3D), oxidoreductase activity (GO:0051903, GO:0004022, GO:0050607) and threonine-type peptidase activity (GO:0004298, GO:0070003) were the most significantly enriched. NAD binding (GO:0051287) and coenzyme binding (GO:0050662) were also enriched. In the CC category (Figure 3E), the cytoplasmic proteasome complex (GO:0031597, GO:0000502, GO:1905369) and plasma membrane components (GO:0005886, GO:0044459, GO:0031226) showed the most significant enrichment.

KEGG pathway analysis further revealed the affected biological networks (Figure 3F). The most significantly enriched pathways included the proteasome pathway (ko03050), glutathione metabolism (ko00480), and the peroxisome pathway (ko04146). Other significant pathways included endocytosis (ko04144), fatty acid degradation (ko00071), and DNA replication (ko03030).

## 4 Discussion

This study demonstrates for the first time that exogenous ferrous sulfate (FeSO₄) potently inhibits the *in vitro* growth of *Trichosporon asahii* and induces a ferroptosis-like cell death. This process exhibits typical hallmarks of ferroptosis: dependence on iron ion influx, burst generation of reactive oxygen species (ROS), occurrence of lipid peroxidation, and ultimately, damage to key membrane systems such as mitochondria and the plasma membrane. Transcriptomic analysis further reveals, at a systems level, that FeSO₄ stress triggers extensive reprogramming of the gene expression network in *T. asahii*, with particularly significant changes in redox processes, glutathione metabolism, and the proteasome pathway.

Iron is an essential trace element for life, and its redox activity is a double-edged sword; it participates in crucial metabolism but can also generate ROS via the Fenton reaction and regulate cell death signaling pathways (Bogdan et al., 2016; Ray et al., 2012). Ferroptosis is a form of non-apoptotic cell death driven by iron-dependent lipid peroxidation, discovered in 2012 (Dixon and Stockwell, 2014). Its precise molecular mechanisms, especially the specific role iron plays, remain a central research focus (Linkermann et al., 2014). This study is the first to confirm in *T. asahii* that exogenous Fe^2+^ can trigger ferroptosis-like death, and this process can be reversed by iron chelators and the specific inhibitor Ferrostatin-1. These findings are consistent with reports in *Candida albicans* (Mo et al., 2024) and *Trichophyton rubrum* (Zhang et al., 2023), strongly suggesting that ferroptosis, as a cell death program, may be conserved among pathogenic fungi.

Our research indicates that exogenous Fe^2+^ rapidly influxes and accumulates intracellularly, and the iron chelator 2,2′-bipyridyl can completely block the lethal effect of FeSO₄, confirming the iron dependence of this process. Notably, FeSO₄ treatment led to significant downregulation of several iron uptake-related genes, including the high-affinity iron permease FTR1 (ncbi_25985289) and siderophore uptake transporters (ncbi_25989769, ncbi_25983893), indicating that cells attempted to suppress iron ion uptake in response to intracellular iron accumulation. However, under the intense FeSO₄ challenge, these endogenous regulatory mechanisms were insufficient to prevent excessive iron influx, ultimately leading to ferroptosis. Many studies have discussed alterations in genes related to iron ion transport and metabolism, and targeting iron overload-related genes or using iron chelators can effectively inhibit ferroptosis (Tang et al., 2021).

The influx of Fe^2+^ primarily catalyzes the massive production of hydroxyl radicals (·OH) through the Fenton reaction, triggering a burst of ROS. Notably, FeSO₄ exhibited stronger antifungal activity than FeCl₂, which may be explained by the fact that chloride ions can act as hydroxyl radical scavengers and interfere with the Fenton reaction, whereas sulfate ions are relatively inert and do not compromise Fe^2+^-mediated ROS production (Tsuneda et al., 2002). Although the Fenton reaction inhibitor Liproxstatin-1 (Lip-1) could partially reverse the killing effect of FeSO₄, it failed to completely suppress ROS generation, suggesting the existence of other ROS sources such as leakage from the mitochondrial electron transport chain. Subsequently, ROS attack polyunsaturated fatty acids in cell membranes, initiating a chain reaction of lipid peroxidation, which is directly confirmed experimentally by increased MDA content and enhanced C11-BODIPY fluorescence signal. The partial alleviation of cell death by the lipid peroxidation inhibitor GSH further establishes the central role of lipid peroxidation.

Severe lipid peroxidation ultimately leads to the loss of integrity in cellular membrane systems. JC-1 staining showed a significant decrease in mitochondrial membrane potential, aligns with features of ferroptosis observed in fungi, such as reduced or vanished mitochondrial cristae and outer membrane rupture in *Saccharomyces cerevisiae* (Han and Lee, 2024), and is also consistent with reports in mammalian systems (Lyamzaev et al., 2023). indicating that impaired mitochondrial function is a key event in the ferroptosis process. Concurrently, Nile Red staining suggested increased cell membrane permeability accompanied by abnormal accumulation of neutral lipids. These critical damages to membrane systems, corroborated by morphological changes observed via scanning electron microscopy, such as cell surface shrinkage and collapse, collectively constitute the final pathway leading to cell death. Furthermore, ferroptosis is closely related to the functions of the endoplasmic reticulum (via pathways like lipid metabolism, oxidative stress, and calcium homeostasis) and peroxisomes (by regulating ROS balance and iron metabolism) (An et al., 2024; Wang et al., 2023), suggesting that multiple organelles synergistically constitute the regulatory network of ferroptosis.

Transcriptomic sequencing analysis reveals, at a systems level, dramatic alterations in the biological network of *T. asahii* under lethal iron stress. Differentially expressed genes are significantly enriched in redox processes (GO:0055114) and oxidoreductase activity (GO:0051903, GO:0004022, GO:0050607), highly consistent with the aforementioned biochemical phenotypes. The expression of key antioxidant enzyme genes increases sharply. Among them, glutathione peroxidase (GPx, ncbi_25991220) is upregulated with a log₂FC ≈ 13.47, and multiple glutathione S-transferase (GST) genes (ncbi_25985663, log₂FC ≈ 5.83; ncbi_25990279, log₂FC ≈ 5.83, etc.) are also significantly upregulated. These results indicate that cells activate the GSH system to cope with extreme oxidative stress. However, despite the mobilization of a robust and extensive antioxidant defense system, the sustained ROS burst and eventual cell death *in vitro* experiments demonstrate that this defense failed to prevent the accumulation of oxidative damage and the onset of ferroptosis.

This study confirms that the cell death triggered by FeSO₄ in *T. asahii* possesses the core features of ferroptosis (iron dependence, ROS, lipid peroxidation), suggesting a considerable degree of conservation in this cell death program between fungi and mammals. However, the specific execution details may involve important species-specific differences. For instance, the homologs and functions of key positive and negative regulators of mammalian ferroptosis (e.g., ACSL4, GPX4) in fungi remain largely unknown. Furthermore, the significant cell wall structural damage observed in this study is a morphological change unique to fungi compared to animal cells, potentially related to ferroptosis. This may originate from direct ROS attack on cell wall polysaccharides or the inactivation of related synthase systems. Differences in fungal cell membrane lipid composition (rich in ergosterol) compared to mammals (rich in cholesterol and polyunsaturated fatty acids) may also affect the specific substrates and kinetics of lipid peroxidation. Therefore, future research urgently needs to identify specific molecular markers and regulatory networks of fungal ferroptosis.

This study has several limitations. First, the effective *in vitro* antifungal concentration of FeSO₄ identified herein is several orders of magnitude higher than peak plasma iron levels achievable through standard oral supplementation, which typically fall within the micromolar range. Consequently, the primary translational challenge is not the inherent toxicity of FeSO₄, but rather (1) the necessity of achieving locally effective antifungal concentrations at infection sites via topical or targeted delivery, and (2) the need to evaluate the safety and pharmacokinetics associated with such elevated local doses in appropriate *in vivo* infection models. Second, all experiments were conducted *in vitro*; the therapeutic efficacy and *in vivo* safety of FeSO₄ in animal infection models remain to be verified. Third, although transcriptomic analysis has suggested the involvement of several key genes (e.g., homologs of GPx, GST, and FTR1) in ferroptosis, their specific functions require confirmation through genetic approaches such as gene knockout or overexpression. Additionally, whether FeSO₄ exerts synergistic effects with conventional antifungal drugs such as azoles represents a promising avenue worthy of further investigation with potential implications for clinical translation.

The FeSO₄ used in this study is chemically identical to clinically approved oral iron supplements with well-documented safety profiles for systemic human use (Fairweather-Tait, 2001). As a highly soluble iron salt with favorable gastrointestinal absorption, FeSO₄ is regarded as the reference standard for evaluating the bioavailability of other iron formulations (Hallberg et al., 1997). However, systemic administration at doses required for antifungal efficacy is clinically unfeasible due to the risk of iron overload and associated systemic toxicity (Anderson, 2007).

Therefore, the translational potential of FeSO₄ depends on topical or targeted delivery strategies capable of achieving high local concentrations at infection sites (e.g., cutaneous lesions or mucosal colonization by *Trichosporon asahii*), while minimizing systemic exposure and the associated risk of iron overload. Recent studies have provided proof-of-concept for topical delivery strategies involving FeSO₄. For instance, FeSO₄-loaded hydrogels have demonstrated favorable therapeutic efficacy and safety in animal models of MRSA keratitis (Wang et al., 2022) and infected wounds (Wang et al., 2024), confirming the application potential of FeSO₄-based hydrogel formulations in treating local infections. Furthermore, microencapsulation of FeSO₄ with the mucoadhesive polymer carbopol significantly enhances its oral bioavailability and enables sustained drug release, offering potential reference value for application scenarios requiring elevated local mucosal concentrations (e.g., gastrointestinal fungal colonization) (Letha et al., 2024).

Future research should focus on: (1) constructing genetically manipulated strains of key genes to validate their functions at the molecular level; (2) establishing animal infection models of *T. asahii* to evaluate the *in vivo* efficacy of ferroptosis inducers; (3) screening and developing more efficient and specific fungal ferroptosis inducers or sensitizers; and (4) assessing the efficacy, local tissue toxicity, and pharmacokinetics of FeSO₄ in murine cutaneous or subcutaneous infection models, while exploring formulation strategies such as hydrogels, creams, or sustained-release devices to enable effective local delivery. Such investigations are essential to determine whether the *in vitro* antifungal activity of FeSO₄ can be translated into safe and effective clinical interventions.

## 5 Conclusion

In summary, this study systematically confirms that exogenous Fe^2+^ can effectively kill the drug-resistant pathogenic fungus *Trichosporon asahii* by inducing ferroptosis-like cell death and preliminarily elucidates its molecular mechanism of iron accumulation—ROS burst—lipid peroxidation—membrane damage. This not only expands our understanding of programmed cell death mechanisms in fungi but, more importantly, proposes ferroptosis as a novel strategy against drug-resistant fungal infections. It provides a significant theoretical basis and new avenues for the development of antifungal drugs with novel mechanisms of action.

## Data Availability

The datasets generated and analyzed during the current study are available in the NCBI BioProject database under the accession number PRJNA1432677.

## Footnotes

1
http://www.geneontology.org/

## Supplementary material

Supplementary Figure S1 Antifungal activity of different iron compounds against *Trichosporon asahii*.
