# Supplementary figures and images for: Ferrous sulfate induces ferroptosis-like cell death in Trichosporon asahii
Source: Front Microbiol. 2026 Apr 1;17:1789479. doi: 10.3389/fmicb.2026.1789479 (PMC13082251; doi:10.3389/fmicb.2026.1789479)

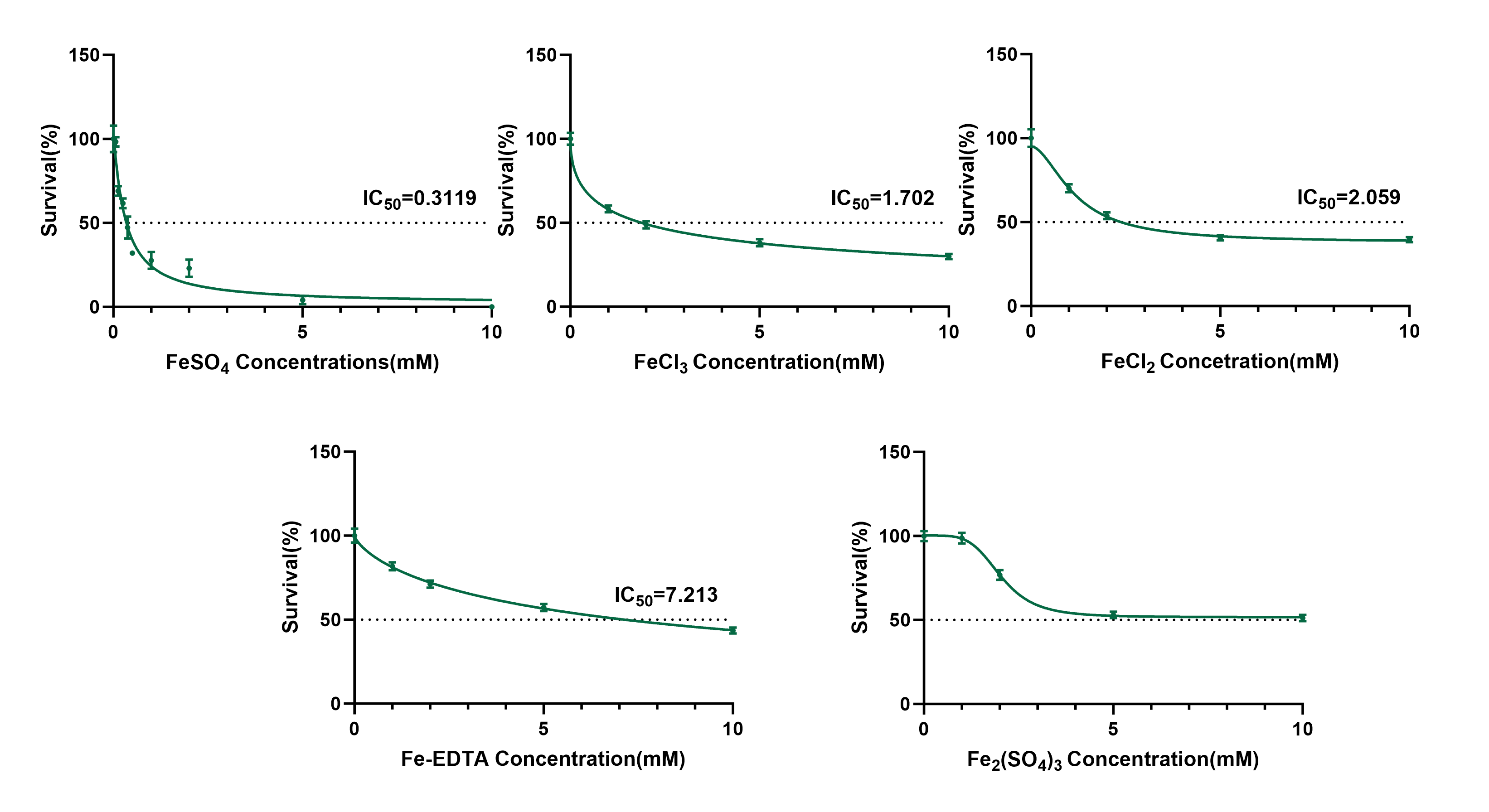

Supplement: Supplementary Figure S1 — Antifungal activity of different iron compounds against Trichosporon asahii. [file Image_1.TIF]
