# Supplementary material for: Ferrous sulfate induces ferroptosis-like cell death in Trichosporon asahii
Source: Front Microbiol. 2026 Apr 1;17:1789479. doi: 10.3389/fmicb.2026.1789479 (PMC13082251; doi:10.3389/fmicb.2026.1789479)
Supplement: Supplementary file 2 [file Table_1.DOCX]

Table S1 Antifungal Susceptibility Profiles of the 11 Tested *T. asahii* Strains

| **Strain** | **FLC**  **(MIC₅₀, μg/mL)** | **AmB**  **(MIC₁₀₀, μg/mL)** | **VRC**  **(MIC₅₀, μg/mL)** | **Clinical source** |
| --- | --- | --- | --- | --- |
| CBS 2479 | 2 | 2 | 0.06 | Nail clippings |
| BMT 06-3-01 | 2 | 2 | 0.06 | Urine |
| BMT 06-3-02 | 2 | 2 | 0.06 | Sputum |
| BMT 06-3-03 | 1 | 1 | 0.03 | Oral cavity |
| BMT 06-3-04 | 2 | 2 | 0.06 | Nail clippings |
| BMT 06-3-05 | 1 | 4 | 0.03 | Sputum |
| BMT 06-3-06 | 2 | 4 | 0.125 | Urine |
| BMT 06-3-07 | 0.5 | 0.5 | 0.03 | Nail clippings |
| BMT 06-3-08 | 2 | 2 | 0.125 | Derma |
| BMT 06-3-09 | 4 | 4 | 0.25 | Urine |
| BMT 06-3-10 | 1 | 2 | 0.03 | Derma |
